# Healthcare Professional Communication on Sexual Health: A Report from the Italian Working Group on Adolescents and Young Adults with Cancer

**DOI:** 10.1093/oncolo/oyad175

**Published:** 2023-06-22

**Authors:** Paola Quarello, Angela Toss, Paola Berchialla, Maurizio Mascarin, Matteo Lambertini, Marta Canesi, Giuseppe Maria Milano, Lorena Incorvaia, Giuseppe Luigi Banna, Fedro Peccatori, Andrea Ferrari

**Affiliations:** Pediatric Onco-Hematology, Stem Cell Transplantation and Cellular Therapy Division, Regina Margherita Children’s Hospital, Torino, Italy; Department of Sciences of Public Health and Pediatrics, University of Turin, Turin, Italy; Department of Oncology and Hematology, Azienda Ospedaliero-Universitaria di Modena, Modena, Italy; Department of Medical and Surgical Sciences, University of Modena and Reggio Emilia, Modena, Italy; Department of Clinical and Biological Sciences, University of Torino, Torino, Italy; AYA Oncology and Department of Radiation Oncology, Centro di Riferimento Oncologico, Aviano, Italy; Departament of Medical Oncology, U.O. Clinica di Oncologia Medica, IRCCS Ospedale Policlinico San Martino, Genova, Italy; Department of Internal Medicine and Medical Specialties (DiMI), School of Medicine, University of Genova, Genova, Italy; Department of Pediatrics, University of Milano-Bicocca, Fondazione IRCCS San Gerardo dei Tintori, Monza, Italy; Department of Pediatric Onco-Hematology and Cell and Gene Therapy, Bambino Gesù Children Hospital, Roma, Italy; Department of Surgical, Oncological and Oral Sciences Section of Medical Oncology University of Palermo, Palermo, Italy; Portsmouth Hospitals University NHS Trust, Portsmouth, PO6 3LY, UK; Gynecologic Oncology Department, European Institute of Oncology IRCCS, Milano, Italy; Pediatric Oncology Unit, Fondazione IRCCS Istituto Nazionale dei Tumori, Milano, Italy

**Keywords:** adolescents and young adults, sexual health, education

## Abstract

**Background:**

Sexual function is an important concern for adolescent and young adult (AYA) with cancer. The aim of this study was to explore the attitude of Italian health care professionals who deal with AYA patients with cancer toward sexual health communication.

**Materials and Methods:**

A 11-question survey was developed by the AIOM (Associazione Italiana di Oncologia Medica) and AIEOP (Associazione Italiana Ematologia Oncologia Pediatrica) AYA workgroup and sent to AIOM and AIEOP members.

**Results:**

The sample comprised 360 respondents, 54.2% AIEOP and 45.8% AIOM members. Eighty percent were physicians, 14.5% nurses, 4.7% psychologists, and 0.8% other professionals. Medical oncologists are more used to investigate about AYA sexual health than pediatric oncologists (58.2% vs. 46.2%), even if pediatrics more frequently refer patients to specific and shared protocol (40% vs. 26.1%). Both AIOM and AIEOP participants mostly talk about sexual health only on request or occasionally (78.8% and 79%, respectively). Clinician-reported barriers to communication identified in this study are lack of preparation and embarrassment for both the categories, plus the presence/interference of parents for pediatrics and lack of time for medical oncologists. Overall, less than 5% of clinicians in our survey received specific training on potential sexual health issues in AYA patients with cancer and only 2% felt adequately prepared to speak about it.

**Conclusion:**

Sexual health is a key component of comprehensive care for AYA with cancer during treatments. This study highlighted the need of Italian providers for specific training and guidelines on sex-related health issues encountered by AYA patients.

## Teaser

Sexual function is a key aspect of quality of life among adolescent and young adult patients with cancer. This article explores attitudes of healthcare professionals toward sexual health communication, identifying barriers to these conversations and evaluating the need for additional education for providers and resources or referrals for patients.

**Implications for Practice**

The identification of sexual concerns and the ability to provide effective interventions may lead to improved clinician and patient comfort talking and facing sexual issues. A multidisciplinary approach between medical and pediatric professionals is key to develop shared recommendations and answer to the need for education and training of health care providers.

## Introduction

Sexual function is a key aspect of quality of life among adolescent and young adult (AYA) patients with cancer.^1,2^ Indeed, a cancer diagnosis and the associated oncological treatments may impact on sexual health at physical, psychosocial, and developmental level. Cancer may threaten sexual identity in several different ways: physical changes, impaired fertility, self-esteem, sexuality, and partnerships.^3-5^ Particularly, women may face early onset of menopause, dyspareunia, lubrication problems, and vaginal stenosis, whereas men may complain erectile and ejaculatory dysfunction. Overall, both sexes may be affected by reduction of libido, loss of desire and satisfaction, orgasmic problems, loss of desire and libido, fatigue, and infertility.^6^

In AYA patients, body image, romantic affection, and sexual function are connected in a complex relationship, and challenges in one area may affect another.^7^ As these issues can negatively impact quality of life, there is an urgent need to help healthcare providers discussing these themes early and provide supportive interventions to address these challenges.^8, 9^ Nevertheless, despite the prevalence of sexual dysfunction in this population, clinicians often underestimate the relevance of psychosexual issues among AYA and do not discuss sexual and reproductive health through disease treatment and survivorship.^10^ Moreover, they report a lack of experience discussing sexual issues and recognize the need for further education regarding sexual health communication.^11^ Lack of knowledge and resources, low priority, parents/family, patient and clinician discomfort, limited time, and lack of rapport have been identified as barriers to communication.^11^

In April 2021, the Italian adult and pediatric oncology societies—AIOM (Associazione Italiana di Oncologia Medica) and AIEOP (Associazione Italiana Ematologia Oncologia Pediatrica)—joined in a Working Group dedicated to AYA, with the aims of increasing awareness among the scientific community, exchanging knowledge, and foreseeing integrated programs to improve the standard of care for AYA with cancer in Italy.^8,12^ One of the first research initiatives of this group focuses on sexual issues that affect AYA patients during and after cancer treatments. Particularly, the present study aimed to explore the attitude of Italian health care professionals who deal with AYA patients with cancer toward sexual health communication, identifying barriers to these conversations and evaluating the need for additional education for providers and resources or referrals for patients.

## Materials and Methods

Study participants were adult and pediatric professionals who are members of AIOM and AIEOP.

Members of the AIEOP-AIOM AYA working group developed the survey content that was reviewed by one adult and one pediatric psychologist prior to administration. The survey included 11 questions regarding participants’ demographics (3), current practices in sexual issue communication (4), barriers to communication (2), and education in addressing sexual issue (2).

The survey was administrated via Google Surveys. Through the official newsletters, survey links were sent to all AIEOP and AIOM members (1176 and 2473 members, respectively). Question format included multiple choice and Likert scale.

The survey remained open for four months. Participant personal information was confidential and was not associated with survey response. Request for ethical committee approval was not sought and consent for this survey not applied considering the minimal risk for individuals for the following reasons: no data through intervention or interaction with the individuals or identifiable private information were obtained. Furthermore, it was a sample survey conducted within the AIEOP and AIOM community with the goal of identifying areas for improvement within the community.

Continuous variables were expressed as the mean ± standard deviation (SD) or median and interquartile range (IQR) when appropriate, while categorical variables were reported as frequency and percentage. The Mann-Whitney or Student’s *t* test, Chi-square and Fisher’s exact tests were used to compare continuous and categorical variables, as appropriate. The significance level was set at *P* < .05. Statistical analysis was performed using R version 4.1.2.

## Results

### Survey Participants

The sample comprised 360 respondents, 195 (54.2%) AIEOP and 165 (45.8%) AIOM members. Eighty percent (288/360) were physicians, 14.5% (52/360) nurses, 4.7% (17/360) psychologists, and 0.8% (3/360) other professionals. 64.7% of respondents were female (233/360). Participants’ ages ranged from 24 to 70.5 years, with a median age of 39.5 years. Among these, 53.6% (193/360) were under 40 at the time of the survey, while 46.4% (167/360) were older than 40. 61.4% (221/360) of participants take care of more than 10 AYAs per year. AIOM survey respondents were nearly all medical oncologists (95.8% vs 66.7%; *P <* .001), and they were more frequently male (43.6% vs 28.2%) and younger than 40 years (74.5 vs 35.8%; *P <* .001) compared to AIEOP respondents (Table 1).

**Table 1.** Demographic characteristics and clinical variables of the health care providers.

	AIOM		AIEOP	%	P	Total	%
	*N*	%	*N*			*N*	
	165	45.8	195	54.2		360	100.0
Professional type	165	100.0	195	100.0	<.001	360	100.0
Medical/pediatric Oncologist	158	95.8	130	66.7		288	80
Nurse	6	3.6	46	23.6		52	14.5
Psychologist	1	0.6	16	8.2		17	4.7
Other	0	0.0	3	1.5		3	0.8
Gender	165	100.0	195	100.0	.003	360	100.0
Male	72	43.6	55	28.2		127	35.3
Female	93	56.4	140	71.8		233	64.7
Age	165		195		<.001	360	100
<40 years	123	74.5	70	35.8		193	53.6
>40 years	42	25.5	125	64.2		167	46.4
Center activity volume	165	100.0	195	100.0	<.001	360	100.0
<10 AYAs/year	89	53.9	50	25.6		139	38.6
>10 AYA/year	76	46.1	145	74.4		221	61.4

Abbreviation: AYA, adolescent and young adult.

### Communication Practices

Among respondents, 51.8% (186/360) reported that they discuss sexual health with AYAs during treatment. Comparing AIEOP and AIOM respondents, the percentage was significantly lower among the former (46.2% vs 58.2%, *P* = .03).

For 69.4% (250/360) of respondents there is no defined management or standard referral pathway if sexual problems occur during treatment or follow-up.

AIEOP respondents were more like to report the presence of a defined management protocol in case of sexual problems in comparison to AIOM respondents (40% vs 26.1%, respectively; *P* = .007) (Table 2).

**Table 2.** Communication practices.

	AIOM	AIEOP	P
	*N* (%)	*N* (%)	
Is it a routine at your center to investigate about adolescent patients’ sexual health at some point during the treatment?			
Yes	96 (58.2%)	90 (46.2%)	.03
If a sexuality issue arises during therapy or follow-up, is there a specific and shared protocol to which the patient should be referred to?			
Yes	43 (26.1%)	78 (40%)	.007
Who do you think should talk to the patient about sexual health issues?			<.001
Multidisciplinary team	133 (80.6%)	28 (14.35%)	
Oncologist	13 (7.8%)	53 (27.1%)	
Psychologist	11 (6.66%)	79 (40.5%)	
Sexologist	7 (4.24%)	32 (16.4%)	
Nurse	0 (0)	2 (1%)	
Parent	1 (0.6%)	1 (0.5%)	

About 44.7% (161/360) of responders reported that a multidisciplinary team should take care primary responsibility for addressing sexual issues in AYAs.

### Communication Barriers

Most of AIEOP and AIOM respondents (78.9%; 284/360) discuss sexual health with AYAs patients only occasionally or after a specific request. 6.7% (24/360) reported that they never discuss sexual health with their patients. AIOM professionals and responders over the age of 40 years discuss more frequently sexual health in comparison to AIEOP and participants under the age of 40 years (Table 3).

**Table 3.** Communication barriers.

	AIOM	AIEOP	*P*	Male	Female	*P*	<40 years	>40 years	P
	*N* (%)	*N* (%)		*N* (%)	*N* (%)		*N* (%)	*N* (%)	
How frequently do you talk to your patients about their sexual health?			*.001*			NS			<.001
Always	32 (19.4%)	20 (10.8%)		6 (4.7%)	33 (14.2%)		21 (10.9%)	31 (18.5%)	
Occasionally	60 (36.4%)	75 (38.5%)		53 (41.7%)	82 (35.2%)		59 (30.6%)	76 (45.5%)	
Only on request	70 (42.4%)	79 (40.5%)		49 (38.6%)	100 (42.9%)		98 (50.8%)	51 (30.5%)	
Never	3 (1.8%)	21 (10.8%)		6 (4.7%)	18 (7.7%)		15 (7.8%)	9 (5.3%)	
What factors do you believe make it challenging to discuss sexuality with the patient?									
Lack of preparation	103 (62.4%)	111 (56.9%)	*.341*	62 (48.8%)	152 (65.2%)	*.004*	128 (66.3%)	86 (51.5%)	.005
Professional discomfort	37 (22.4%)	45 (23.1%)	*.983*	32 (25.2%)	50 (21.5%)	*.499*	53 (27.5%)	29 (17.4%)	.02
Concern over embarrassing patients	94 (57%)	104 (53.3%)	*.559*	71 (55.9%)	127 (54.5%)	*.885*	125 (64.8%)	73 (43.7%)	<.001
Presence/interference of a parents	51 (30.9%)	93 (47.7%)	*.002*	54 (42.5%)	90 (38.6%)	*.543*	74 (38.3%)	70 (41.9%)	.52
Lack of time	59 (35.8%)	36 (18.5%)	*<.001*	37 (29.1%)	58 (24.9%)	*.455*	59 (30.6%)	36 (21.5%)	.056
None	7 (4.2%)	12 (6.2%)	*.568*	7 (5.5%)	12 (5.2%)	*1.000*	3 (1.6%)	16 (9.6%)	.0006

Prevalent barriers reported in discussing sexual health included concern over embarrassing patients (28.3%), lack of preparation (26%), and presence/interference of parents (19.4%). The lack of time or discomfort was reported as minor constrains (12.6% and 11.1%, respectively).

When compared to male participants, female professionals reported lack of preparation more frequently (65.2% vs 48.8%, *P* = .004). Respondents under the age of 40 reported these issues more frequently than those over the age of 40: lack of preparation (66.3% vs. 51.5%, *P* = .005), discomfort (27.5% vs. 17.4%, *P* = .02), and concern over embarrassing patients (64.8% vs. 43.7%, *P <* .001).

### Education

A total of 66.1% (238/360) of respondents reported that they were either a little or completely unprepared to talk about sexual health. Only 31.9% (115/360) of respondents referred to be quite well prepared.

No differences were found between AIEOP and AIOM professionals, as well as between male and female respondents. Professionals over the age of 40 reported to be better prepared than those under 40 (Table 4).

**Table 4.** Education.

	AIOM	AIEOP	*P*	Male	Female	*P*	<40 years	>40 years	*P*
	*N* (%)	*N* (%)		*N* (%)	*N* (%)		*N* (%)	*N* (%)	
How prepared do you feel to discuss sexual health with an AYA patient?			.101			*.356*			.0002
Well prepared	3 (1.8%)	4 (2.1%)		3 (2.4%)	4 (1.7%)		4 (2.1%)	3 (1.8%)	
Somewhat prepared	45 (27.3%)	70 (35.9%)		47 (37%)	68 (29.2%)		42 (21.8%)	73 (43.7%)	
A little prepared	105 (63.6%)	99 (50.8%)		64 (50.4%)	140 (60.1%)		125 (64.8%)	79 (47.3%)	
Not at all	12 (7.3%)	22 (11.3%)		13 (10.2%)	21 (9%)		22 (11.4%)	12 (7.2%)	
Have you received specific training on potential sexual health issues in AYA patients with cancer?			.519			.794			.5
Yes	6 (3.6%)	11 (5.6%)		7 (5.5%)	10 (4.3%)		7 (3.6%)	10 (5.9%)	

The majority of respondents (95.3%; 343/360) reported that they did not received a specific training on sexual health in AYA patients. No differences related to age, gender of pediatric, or adult professionals were reported.

## Discussion

The present study evaluates attitudes and experiences of pediatric and medical oncology providers toward sexual health communication with AYA patients with cancer. The survey explored communication practices, barriers and need for specific education. Overall, most of the AIOM professionals that answered the survey were medical doctor (95.8%), female (56.4%), and under the age of 40 (74.5%). In addition, just half of respondents declared to treat more than 10 AYA patients/year. On the other hand, although most of pediatric oncology providers are clinicians (66.7%) and female (71.8%) as well, a high rate of nurses answered the survey (23.6%), likely highlighting a higher involvement of these figures in the activities of their society. Contrary to AIOM respondents, most of AIEOP participants are >40 years (64.2%) and treat more than 10 AYA patients/year (74.4%). Participant response rate is lower than desired in this survey (6.6% of AIOM members and 16.5% of AIEOP members), and only for pediatric oncology providers it falls within the range of physician response rates observed in previous survey studies (12%-50%).^13^

Overall, medical oncologists are more used to investigate about AYA sexual health than pediatric oncologists (58.2% vs. 46.2%), even if pediatrics more frequently refer patients to specific and shared protocol (40% vs. 26.1%). Interestingly, whereas AIOM participants allocated the responsibility for discussing sexual health to a multidisciplinary team, pediatrics preferred that psychologists and clinicians should play the central role. A previous qualitative study showed that a complementary team approach, with clearly defined roles for different team members, is required to improve communication about sexual health in patients with cancer.^14^ Identifying team members, such as nurses, social workers, psychologists, sexologists, physician assistants, and physical therapists who have the proper experience and skills to communicate about sexual health with AYAs, may improve the likelihood of counselling is offered, and reduce the burden of time from the oncologist.^6^

Both AIOM and AIEOP participants mostly talked about sexual health only on request or occasionally (78.8% and 79%, respectively), whereas 19.4% of medical oncologists always talk about these issues compared to 10.8% of pediatrics. This means that patient-clinician conversations on sexual issues take place infrequently, although AYA patients consistently identify the need for improved communication on these subjects^15,16^ and despite the recommendations of the American Academy of Pediatrics (AAP), the National Comprehensive Cancer Network (NCCN), and the American Society of Clinical Oncology (ASCO).^9,17,18^

Clinician-reported barriers to communication identified in this study are lack of preparation and embarrassment for both the categories, plus the presence/interference of parents for pediatrics and lack of time for medical oncologists. In particular, lack of preparation is more commonly reported by female clinicians and young colleagues; young clinicians also more frequently report professional discomfort and concern over embarrassing patients. These barriers are similar to those reported by clinicians in previous experiences in both pediatric and adult cancer populations.^11,19^

To facilitate discussing sexual health, clearly defined responsibilities within the team and sufficient knowledge are important.^20^ Prior studies indicate that allowing time for AYAs to speak to their health providers alone and in a protected environment would offer more opportunities to develop relationships and ask questions, especially around more sensitive topics areas such as sexual health. Moreover, in a previous survey, 50% of pediatric oncologists expressed the need for further education on sexual function and gender identity/sexual orientation, and more than 30% reported a need for more education on body image, sexual activity/safe sex practices, and contraception.^6^ On the other hand, several studies demonstrated an overall lack of medical knowledge about LGBTQ patient health care and highlighted the need for more education among oncology healthcare providers.^21-24^ Improving clinician knowledge on gender identities and sexual orientation and how sexual health needs may differ is an important step in ensuring all conversations are inclusive and may reduce professional discomfort during sexual health communication.

Overall, less than 5% of clinicians in our survey received specific training on potential sexual health issues in AYA patients with cancer and only 2% felt adequately prepared to speak about it. Interestingly, this do not significantly differ between pediatrics and oncologists. Most of participants in the survey declared to be inadequately prepared to discuss sexual health with AYA patients, and this was particularly felt by women and younger colleagues. Previous research identified some facilitating strategies to improve sexual health communication, including self-reported questionnaire for the patients, material to hand out, a checklist for healthcare providers, use of a notification to prepare patients prior to conversations, screening tools, and establishing a relationship prior to the conversations.^20,25^ Furthermore, several evidence-based strategies have been developed to guide clinicians through sexual health conversations, such as the 5 As (Ask, Advise, Assess, Assist, and Arrange) communication model and the extended PLISSIT or 5 Ps models.^17,26,27^ These models start by guiding the clinician to introduce the topic and ask the patient for permission to proceed with the conversation. They proceed providing patients with a brief overview on a specific sexual health topic and then, the clinician asks the AYA additional questions to understand his or her education and support needs. This is followed by provision of brief counseling and/or making specialistic referrals (urology, gynecology, reproductive endocrinology, adolescent medicine, psychology, etc.). Finally, the clinician schedules follow-up visits to ensure that the problems have been addressed.^6^

To conclude, sexual health is a key component of comprehensive care for AYA with cancer during treatments. Providers caring for these patients should understand how cancer treatment may negatively impact on sexual health and learn the skills to discuss and address sexual health issues. The results of our survey highlights the need of Italian providers for specific training and guidelines on sex-related health issues encountered by AYA patients, including effective communication strategies to facilitate conversation, fertility risk and preservation strategies, safe sex practices during therapy, gender identities and sexual orientation, contraception, and risk for sexually transmitted diseases. A clear understanding of how to initiate sexual health conversations, the identification of sexual concerns and the ability to provide effective interventions may lead to improved clinician and patient comfort talking and facing sexual issues.

The Italian Working Group on AYA is now working on a survey for patients, together with the main Italian associations involved in sexual health. The multidisciplinary approach is key to develop shared recommendations and answer to the need for education and training of healthcare providers.

## Data Availability

The data underlying this article will be shared on reasonable request to the corresponding author.

## References

[1] Pulewka K , Strauss B, Hochhaus A, Hilgendorf I. Clinical, social, and psycho-oncological needs of adolescents and young adults (AYA) versus older patients following hematopoietic stem cell transplantation. J Cancer Res Clin Oncol. 2021;147(4):1239-1246. 10.1007/s00432-020-03419-z. 33052515 PMC7954716

[2] Wettergren L , Kent EE, Mitchell SA, et al; AYA HOPE Study Collaborative Group. Cancer negatively impacts on sexual function in adolescents and young adults: the AYA HOPE study. Psychooncology. 2017;26(10):1632-1639. 10.1002/pon.4181. 27240019 PMC7239373

[3] Mütsch J , Friedrich M, Leuteritz K, et al. Sexuality and cancer in adolescents and young adults - a comparison between reproductive cancer patients and patients with non-reproductive cancer. BMC Cancer. 2019;19(1):828. 10.1186/s12885-019-6009-2. 31438895 PMC6704507

[4] Saris LMH , Vlooswijk C, Kaal SEJ, et al. A negative body image among Adolescent and Young Adult (AYA) Cancer Survivors: results from the population-based SURVAYA study. Cancers (Basel). 2022;14(21):5243-5243. 10.3390/cancers14215243. 36358662 PMC9655157

[5] Reinman L , Coons HL, Sopfe J, Casey R. Psychosexual Care of Adolescent and Young Adult (AYA) cancer survivors. Children (Basel) 2021;8(11):1058-1058. 10.3390/children8111058. 34828771 PMC8618923

[6] Frederick NN , Lehmann V, Ahler A, et al. Psychosexual functioning in cancer survivorship: what the pediatric oncologist needs to know. Pediatr Blood Cancer. 2021;e28437:e28437. 10.1002/pbc.28437. PMC9167888 34873822

[7] Cherven B , Sampson A, Bober SL, et al. Sexual health among adolescent and young adult cancer survivors: a scoping review from the Children’s Oncology Group Adolescent and Young Adult Oncology Discipline Committee. CA Cancer J Clin. 2021;71(3):250-263. 10.3322/caac.21655. 33283888 PMC8678924

[8] Ferrari A , Quarello P, Mascarin M, et al. Italian pediatric and adult oncology communities join forces for a national project dedicated to adolescents and young adults with cancer. Tumori. 2022;108(2):104-110. 10.1177/03008916211058790. 34841968

[9] Carter J , Lacchetti C, Andersen BL, et al. Interventions to address sexual problems in people with cancer: American Society of clinical oncology clinical practice guideline adaptation of cancer care Ontario guideline. J Clin Oncol. 2018;36(5):492-511. 10.1200/JCO.2017.75.8995. 29227723

[10] Thompson K , Dyson G, Holland L, Joubert L. An exploratory study of oncology specialists’ understanding of the preferences of young people living with cancer. Soc Work Health Care. 2013;52(2-3):166-190. 10.1080/00981389.2012.737898. 23521383

[11] Frederick NN , Campbell K, Kenney LB, et al. Barriers and facilitators to sexual and reproductive health communication between pediatric oncology clinicians and adolescent and young adult patients: the clinician perspective. Pediatr Blood Cancer. 2018;65(8):e27087. 10.1002/pbc.27087. 29697189

[12] Zecca M , Ferrari A, Quarello P, et al. Childhood cancer in Italy: background, goals, and achievements of the Italian Paediatric Hematology Oncology Association (AIEOP). Tumori. 2021;107(5):370-375. 10.1177/03008916211007934. 33876662

[13] Cunningham CT , Quan H, Hemmelgarn B, et al. Exploring physician specialist response rates to web-based surveys. BMC Med Res Methodol. 2015;15(0):32. 10.1186/s12874-015-0016-z. 25888346 PMC4404667

[14] de Vocht H , Hordern A, Notter J, van de Wiel H. Stepped Skills: a team approach towards communication about sexuality and intimacy in cancer and palliative care. Australas Med J 2011;4(11):610-619. 10.4066/AMJ.20111047. 23386876 PMC3562918

[15] Frederick NN , Revette A, Michaud A, Bober SL. A qualitative study of sexual and reproductive health communication with adolescent and young adult oncology patients. Pediatr Blood Cancer. 2019;66(6):e27673. 10.1002/pbc.27673. 30767372

[16] Perez GK , Salsman JM, Fladeboe K, et al. Taboo topics in adolescent and young adult oncology: strategies for managing challenging but important conversations central to adolescent and young adult cancer survivorship. Am Soc Clin Oncol Educ Book. 2020;40(0):1-15. 10.1200/EDBK_279787. PMC7328818 32324424

[17] Marcell AV , Burstein GR, Braverman P, et al. Sexual and reproductive health care services in the pediatric setting. Pediatrics. 2017;140(5):1-13. doi:10.1542/peds.2017-2858. 29061870

[18] Coccia PF , Pappo AS, Beaupin L, et al. Adolescent and young adult oncology, version 2.2018, NCCN clinical practice guidelines in oncology. J Natl Compr Canc Netw. 2018;16(1):66-97. 10.6004/jnccn.2018.0001. 29295883

[19] Park ER , Norris RL, Bober SL. Sexual health communication during cancer care: barriers and recommendations. Cancer J. 2009;15(1):74-77. 10.1097/PPO.0b013e31819587dc. 19197178

[20] Albers LF , Bergsma FB, Mekelenkamp H, et al. Discussing sexual health with adolescent and young adults with cancer: a qualitative study among healthcare providers. J Cancer Educ. 2022;37(1):133-140. 10.1007/s13187-020-01796-0. 32557400 PMC8816785

[21] Banerjee SC , Walters CB, Staley JM, Alexander K, Parker PA. Knowledge, beliefs, and communication behavior of oncology Health-care Providers (HCPs) regarding Lesbian, Gay, Bisexual, and Transgender (LGBT) patient health care. J Health Commun. 2018;23(4):329-339. 10.1080/10810730.2018.1443527. 29521575 PMC5961501

[22] Banerjee SC , Staley JM, Alexander K, Walters CB, Parker PA. Encouraging patients to disclose their lesbian, gay, bisexual, or transgender (LGBT) status: oncology health care providers’ perspectives. Transl Behav Med. 2020;10(4):918-927. 10.1093/tbm/iby105. 30476333 PMC7543074

[23] Shetty G , Sanchez JA, Lancaster JM, et al. Oncology healthcare providers’ knowledge, attitudes, and practice behaviors regarding LGBT health. Patient Educ Couns. 2016;99(10):1676-1684. 10.1016/j.pec.2016.05.004. 27161166 PMC5527558

[24] Pecoriello J , Sampson A, Block R, et al. For the LOvE of reproductive health communication: assessment of the LGBT Oncofertility Education (LOvE) Module. J Adolesc Young Adult Oncol 2022. 10.1089/jayao.2022.0118. PMC10457611 36459104

[25] Sopfe J , Marsh R, Frederick NN, et al. Adolescent and young adult childhood cancer survivors’ preferences for screening and education of sexual function. Pediatr Blood Cancer. 2021;68(12):e29229. 10.1002/pbc.29229. 34245209

[26] Bober SL , Reese JB, Barbera L, et al. How to ask and what to do: a guide for clinical inquiry and intervention regarding female sexual health after cancer. Curr Opin Support Palliat Care 2016;10(1):44-54. 10.1097/SPC.0000000000000186. 26716390 PMC4984532

[27] Taylor B , Davis S. Using the extended PLISSIT model to address sexual healthcare needs. Nurs Stand. 2006;21(11):35-40. 10.7748/ns2006.11.21.11.35.c6382. 17165482

